# Association of hemoglobin glycation index with all-cause and cardio-cerebrovascular mortality among people with metabolic syndrome

**DOI:** 10.3389/fendo.2024.1447184

**Published:** 2024-11-29

**Authors:** Leiyong Zhao, Chengjun Li, Hequn Lv, Chunli Zeng, Yongjun Peng

**Affiliations:** ^1^ Department of Acupuncture and Rehabilitation, Affiliated Hospital of Nanjing University of Chinese Medicine, Nanjing, China; ^2^ Department of Neurology, Huangdao District Hospital of Traditional Chinese Medicine, Qingdao, China

**Keywords:** hemoglobin glycation index, all-cause mortality, cardio-cerebrovascular mortality, cohort study, metabolic syndrome

## Abstract

**Background:**

Research on the association between blood glucose-related biomarkers and mortality has gained increasing attention. However, the association of hemoglobin glycation index (HGI) with all-cause and cardio-cerebrovascular mortality among people with metabolic syndrome has never been investigated. The objective of this study was to examine the association through a cohort study of the American population.

**Methods:**

In this study, 8,267 participants were included. We utilized multivariable Cox regression analyses to explore the relationship between HGI and outcomes. The dose-response relationship between HGI and mortality was explored with restricted cubic splines. Recursive algorithms and segmented linear regression models were used to calculate the inflection points and assess the effect relationships before and after the inflection points.

**Results:**

In the model adjusting for all covariates, our analysis did not reveal a statistically significant association between HGI and mortality. Intriguingly, subsequent explorations of non-linear relationships unearthed a U-shaped correlation between HGI and both all-cause mortality and cardio-cerebrovascular mortality among American adults with metabolic syndrome. Before and after the inflection point, the HRs (95%CIs) for the association between HGI and all-cause mortality were 0.72 (0.63, 0.82) and 1.30 (1.17, 1.44), respectively. For cardio-cerebrovascular mortality, similar opposite relationships were found. The metabolic syndrome population with HGI levels at T2 had a lower rate of mortality.

**Conclusion:**

This cohort study of the American metabolic syndrome population highlighted a U-shaped association of HGI with all-cause and cardio-cerebrovascular mortality.

## 1 Introduction

In the current field of medical research, the widespread impact of metabolic syndrome has emerged as a significant public health issue (1). Metabolic syndrome comprises a cluster of risk factors including hypertension, high blood glucose, abnormal cholesterol levels, and abdominal obesity, which significantly increase the risk of heart disease, diabetes, stroke, and other health problems (2, 3). According to the World Health Organization, metabolic syndrome affects approximately 25% of the global adult population and is one of the primary drivers of cardiovascular diseases and early mortality (4). While existing research has covered its risk factors and consequences, accurately predicting its associated mortality risks from a biomarker perspective remains an urgent research topic.

The hemoglobin glycation index (HGI) is a novel biomarker that has gained attention in the field of metabolic disease in recent years (5). It assesses the extent of excess glycation of hemoglobin by comparing an individual’s actual glycated hemoglobin (HbA1c) level with the expected level based on their average blood glucose level (6, 7). A high HGI indicates that a person’s hemoglobin glycation exceeds what would be predicted from their average blood glucose levels, suggesting a more severe disturbance in glucose metabolism within the body. Abnormalities in glucose metabolism are closely associated with the development and prognosis of vascular disease (8, 9). Prolonged hyperglycemia can lead to vascular endothelial cell damage and promote the formation of atherosclerosis, thus increasing the risk of cardio-cerebrovascular diseases (10). Research indicates that HGI is significantly associated with microvascular complications and cardiovascular disease risk in diabetic patients (11, 12). Given the strong correlation between diabetes and metabolic syndrome, it is hypothesized that HGI may be able to serve as an independent biomarker for predicting cardio-cerebrovascular mortality in patients with metabolic syndrome.

Currently, the application of the HGI in diabetes management is gradually gaining recognition, yet its role and significance in patients with metabolic syndrome have not been thoroughly researched. Particularly, there is a relative scarcity of studies concerning the relationship between HGI and the risks of cardio-cerebrovascular and all-cause mortality in patients with metabolic syndrome. Early research, focused on diabetic populations, has found that individuals with high HGI are more prone to cardio-cerebrovascular complications (13). Given the complexity of metabolic syndrome and its strong association with various mortality risks, exploring the relationship between HGI and these risks could not only enhance our understanding of the pathophysiology of metabolic syndrome but might also help clinicians more accurately assess and manage health risks in clinical practice. Therefore, this study utilized data from the National Health and Nutrition Examination Survey (NHANES) (1999–2018) to investigate the association between the HGI and the risks of all-cause and cardio-cerebrovascular mortality in population with metabolic syndrome. Through this study, we hope to provide new insights into clinical practice, facilitate the development of public health interventions, and ultimately reduce the risk of mortality in patients with metabolic syndrome.

## 2 Methods

### 2.1 Study population

The current study utilized data derived from the NHANES, a comprehensive cross-sectional survey undertaken under the auspices of the Centers for Disease Control and Prevention in the United States. This survey systematically collects health and nutrition-related data from a representative sample of American adults and children. The protocol for this survey was ethically approved by the Research Ethics Review Board of the National Center for Health Statistics, and informed consent was obtained in writing from all participants.

**Figure 1:**
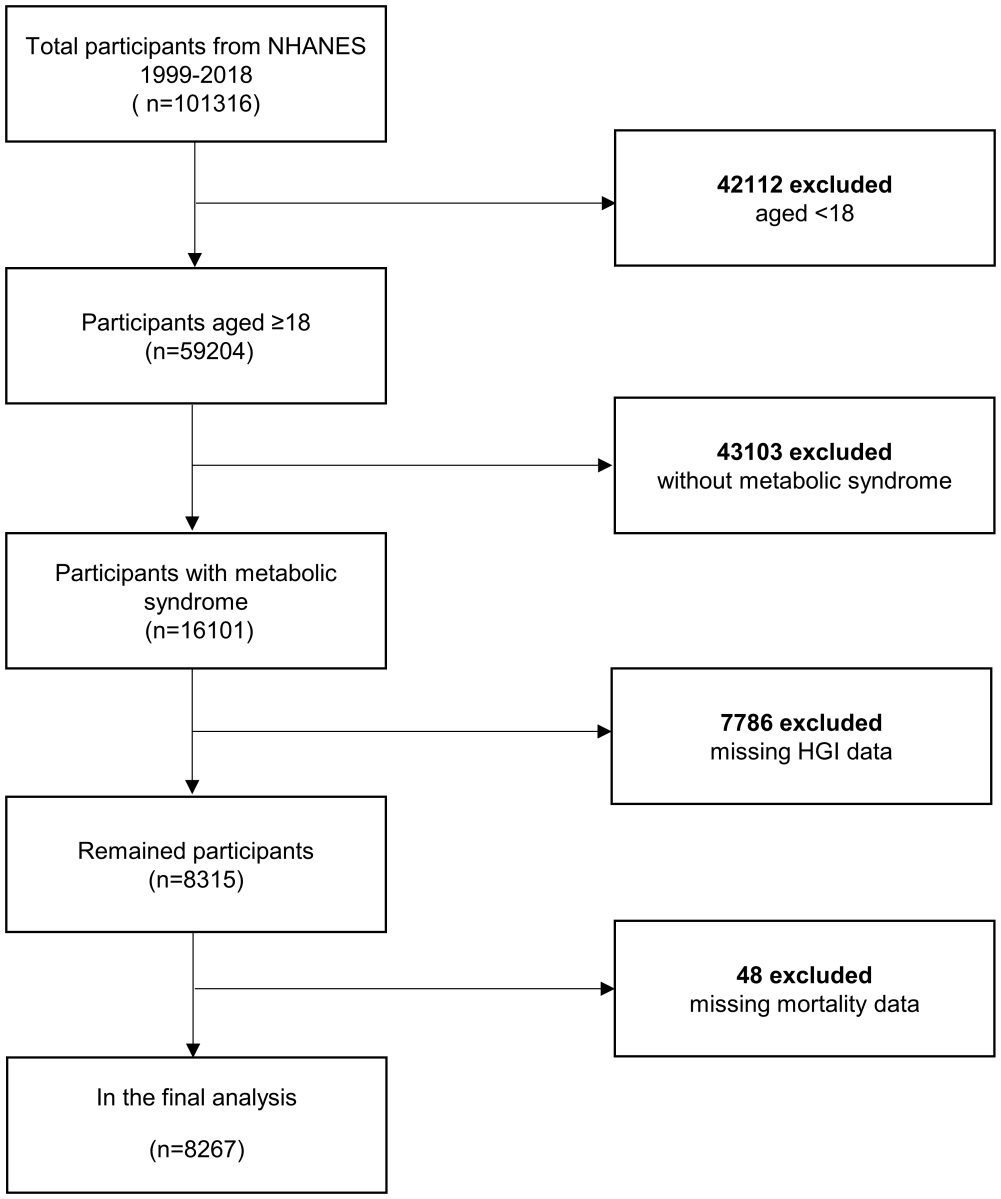
Flow chart of sample inclusion and exclusion in current study.

Our analysis incorporated two decades of adult data from NHANES (1999–2018). Metabolic syndrome in the adult population was delineated according to the NCEP ATP III-2005 benchmarks (3 or more criteria from the following 5 criteria) (14, 15). The criteria comprised (1): a threshold waist circumference (WC) of 102 cm or more for males and 88 cm or more for females (2); systolic/diastolic blood pressure equal to or exceeding 130/85 mmHg, or pharmacological intervention for previously diagnosed hypertension (3); HDL cholesterol concentrations less than 40 mg/dL in males and 50 mg/dL in females (4); triglyceride levels at or above 150 mg/dL, or the utilization of triglyceride-lowering medications; and (5) fasting glucose concentration of 100 mg/L or higher, or employment of insulin or other antihyperglycemic agents. The study cohort was refined by excluding individuals younger than 18 years old, participants who did not have metabolic syndrome, those with missing HGI data, and those lacking complete mortality data, resulting in a final sample of 8,267 individuals (**Figure 1**).

### 2.2 Measurement of hemoglobin glycation index

**Figure 2:**
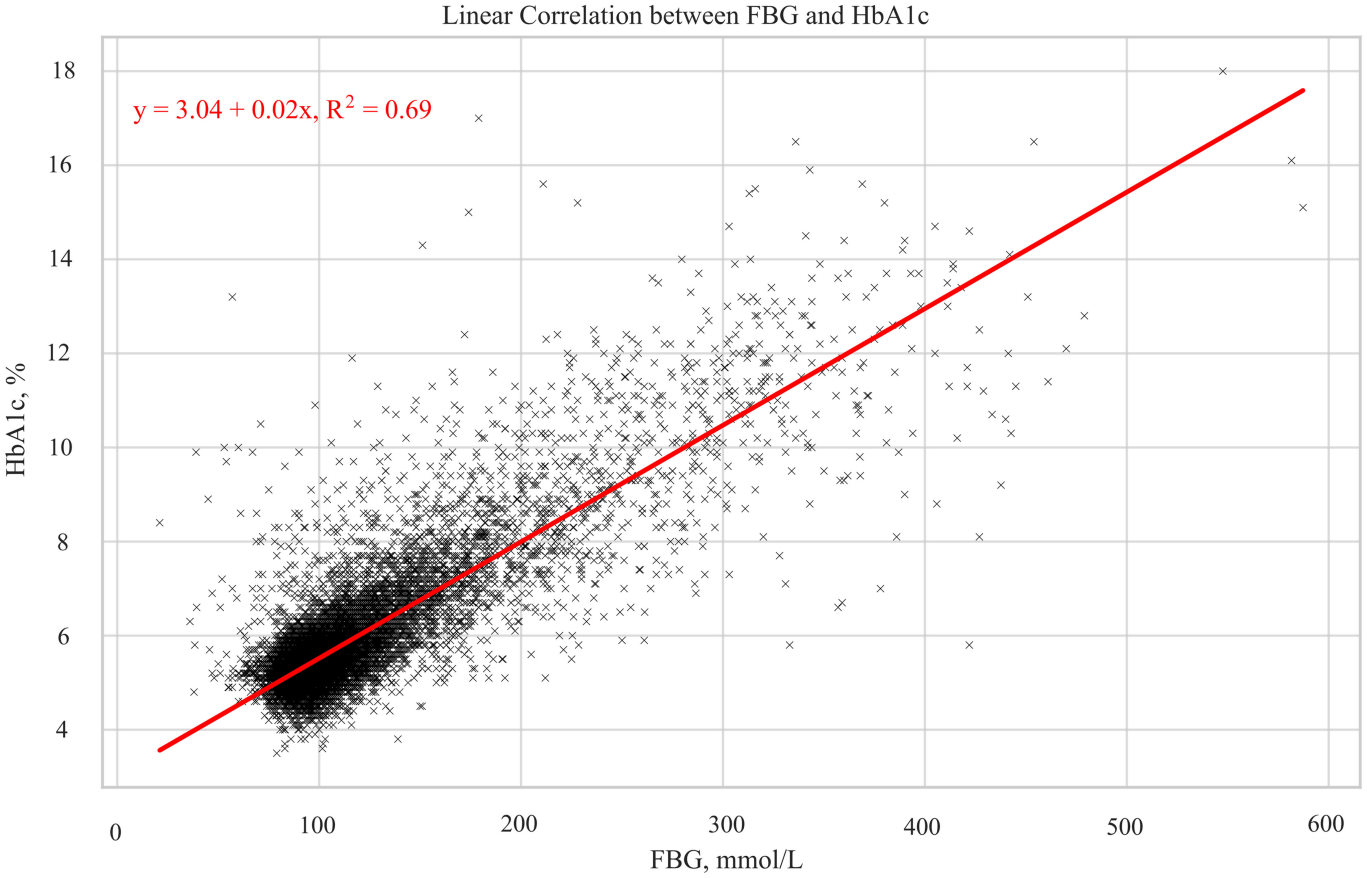
Linear correlation between FBG and HbA1c.

The HGI essentially measured the deviation of an individual’s HbA1c from what would be predicted based on their fasting blood glucose (FBG) level (16). This index was calculated through the formula: HGI = measured HbA1c- predicted HbA1c. The predicted HbA1c is determined by inserting individual FBG levels into a linear regression equation, defined as “Predicted HbA1c = 0.02 × FBG (mg/dL) + 3.04”(**Figure 2**). The data for HbA1c and fasting plasma glucose were derived from biochemical tests. Currently, there is no defined threshold criterion for HGI, so we categorized it into three groups based on the tertiles of its distribution.

### 2.3 Ascertainment of mortality

The study obtained data on all-cause and cardio-cerebrovascular mortality from the National Death Index (NDI), a centralized repository managed by the National Center for Health Statistics that compiles mortality records from across the United States. For this analysis, we utilized the publicly available NHANES-linked mortality file updated as of December 31, 2019, accessible via https://www.cdc.gov/nchs/datalinkage/mortality-public.htm. This file links NHANES participants to NDI records using a sophisticated probabilistic matching algorithm, thereby facilitating accurate ascertainment of mortality status. The definition of mortality causes was on the basis of the International Statistical Classification of Diseases and Related Health Problems, 10th Revision (ICD-10) codes. Specifically, cardio-cerebrovascular mortality was identified through ICD-10 codes corresponding to heart diseases (100–109, 111, 113, 120–151) and cerebrovascular diseases (160–169) (17).

### 2.4 Assessment of covariates

Informed by previous research (18, 19), our study incorporated a diverse array of confounders related to mortality and HGI, encompassing demographic data (age, gender, race/ethnicity, educational level, marital status, and income-to-poverty ratio), lifestyle habits (smoking status, drinking status, and vigorous recreational activities), chronic disease states (hypertension, hyperlipidemia, diabetes, CVD, CKD, and stroke), medication use (anti-hypertensives, anti-diabetics, and anti-hyperlipidemic drugs), and physical and biochemical measurements (body mass index, WC, fasting triglycerides, uric acid, fasting total cholesterol, and C-reactive protein). This comprehensive approach was employed to ensure a nuanced analysis of the relationship between HGI and mortality, taking into account a wide spectrum of potential influencing factors.

### 2.5 Statistical analysis

To represent the general population of the United States, the NHANES employed a stratified, multi-stage probability design. Consequently, we applied weighting in our analysis to adjust for non-randomness in the sample and ensure the broad applicability of the results. The division of study subjects into three groups was based on the HGI tertiles. In describing the baseline characteristics of the population, the representation of continuous variables was in the form of mean values with standard error, whereas categorical variables were depicted as percentages with standard error. To compare the differences among the three groups, one-way ANOVA tests were used for continuous variables, and chi-square tests for categorical variables.

To mitigate the impact of confounding factors on the results, we constructed multivariable Cox proportional hazards regression models. Model 1 did not adjust for any covariates to present the original association. Model 2 adjusted for age, gender, and race, and Model 3 adjusted for all included covariates to control for possible confounding effects, providing a more accurate estimate of the relationship between HGI and risk of mortality. The results were presented as hazard ratios (HRs) and 95% confidence intervals (CIs). To explore the stability of our findings, we conducted linear trend tests across HGI categories to assess whether the results changed linearly as HGI increased. The dose-response association between HGI and mortality was examined using restricted cubic splines with covariate adjustments. Non-linearity was tested using a likelihood ratio test. Upon detecting non-linearity, a recursive algorithm was used to calculate the inflection point, and segmented linear regression models were established to assess the effect relationship before and after the inflection point. Moreover, the Kaplan-Meier (KM) survival analysis was conducted to measure trends in the risk of mortality at different levels of HGI. All analyses were performed using R (version 4.2.1) and Empower Statistics software. A two-tailed *P-value <*0.05 was considered statistically significant.

## 3 Results

### 3.1 Baseline characteristics

The characteristic information of the population was displayed in **Table 1**, categorized by the HGI (T1: < 0.31, n=2756; T2: 0.31-0.70, n=2771; T3: >0.70, n=2740). A total of 8,267 participants were included in this study. The average age was 54.29 (standard error 0.26) years, with males constituting 51.80% and females 48.20%. The racial composition was 71.91% White and 9.05% Black, with 66.60% of participants having an above-high school education level. Compared to other groups, individuals in the highest HGI category (T3) were more likely to be older, have a lower educational level, higher risks of CVD, CKD, and stroke, more frequent use of anti-hypertensive, anti-hyperlipidemic, and anti-diabetic medications, higher body mass index and WC. In biochemical examinations, they had higher levels of HbA1c and C-reactive protein. Furthermore, they exhibited a higher probability of all-cause and cardio-cerebrovascular mortality.

**Table 1:** Baseline characteristics according to HGI category.

Variable	Total (n=8267)	T1 (n=2756) < 0.31	T2 (n=2771) 0.31-0.70	T3 (n=2740) >0.70	P-value
Age (years)	54.29 (0.26)	50.87 (0.43)	54.74 (0.35)	58.71 (0.35)	< 0.0001
Gender (%)					< 0.0001
Male	48.20 (0.02)	55.86 (1.22)	43.69 (1.27)	42.88 (1.29)	
Female	51.80 (0.02)	44.14 (1.22)	56.31 (1.27)	57.12 (1.29)	
Race/ethnicity (%)					< 0.0001
Mexican American	8.02 (0.01)	7.61 (0.69)	8.12 (0.75)	8.50 (0.81)	
White	71.91 (0.03)	78.07 (1.15)	72.72 (1.46)	61.91 (1.83)	
Black	9.05 (0.00)	5.14 (0.41)	7.80 (0.66)	16.36 (1.15)	
Other	11.01 (0.01)	9.18 (0.80)	11.35 (0.91)	13.23 (1.03)	
Education level (%)					< 0.0001
Less than high school	7.56 (0.00)	5.52 (0.42)	7.32 (0.51)	10.83 (0.76)	
High school	25.84 (0.01)	23.70 (1.21)	26.16 (1.15)	28.55 (1.37)	
More than high school	66.60 (0.02)	70.78 (1.25)	66.52 (1.27)	60.62 (1.54)	
Marital status (%)					< 0.0001
Married/Living with partner	65.83 (0.02)	66.65 (1.33)	66.54 (1.28)	63.73 (1.21)	
Divorced/separated/widowed	23.50 (0.01)	20.22 (1.06)	24.00 (1.01)	27.64 (1.08)	
Never married	10.67 (0.01)	13.13 (1.04)	9.46 (0.74)	8.63 (0.70)	
Smoking status (%)					0.35
Now	20.18 (0.01)	19.00 (1.12)	21.31 (0.94)	20.46 (1.15)	
Former	31.57 (0.01)	31.03 (1.18)	31.55 (1.20)	32.38 (1.31)	
Never	48.25 (0.01)	49.98 (1.34)	47.14 (1.26)	47.16 (1.44)	
Drinking status (%)					< 0.0001
Now	66.48 (0.02)	72.61 (1.21)	65.85 (1.26)	58.40 (1.36)	
Former	20.26 (0.01)	17.07 (1.01)	20.89 (1.06)	24.08 (1.10)	
Never	13.26 (0.01)	10.32 (0.77)	13.26 (0.94)	17.53 (1.13)	
Vigorous recreational activities (%)					< 0.001
Yes	60.98 (0.02)	57.54 (1.24)	62.02 (1.31)	64.63 (1.28)	
No	39.02 (0.01)	42.46 (1.24)	37.98 (1.31)	35.37 (1.28)	
Hyperlipidemia (%)					0.02
Yes	5.98 (0.00)	7.22 (0.82)	5.03 (0.52)	5.38 (0.50)	
No	94.02 (0.03)	92.78 (0.82)	94.97 (0.52)	94.62 (0.50)	
Hypertension (%)					< 0.0001
Yes	35.07 (0.01)	40.82 (1.52)	35.82 (1.24)	25.72 (1.15)	
No	64.93 (0.02)	59.18 (1.52)	64.18 (1.24)	74.28 (1.15)	
Diabetes (%)					< 0.0001
Yes	57.76 (0.02)	50.83 (1.31)	48.78 (1.35)	79.44 (1.03)	
No	42.24 (0.01)	49.17 (1.31)	51.22 (1.35)	20.56 (1.03)	
CKD (%)					< 0.0001
Yes	23.63 (0.01)	18.67 (0.99)	21.10 (1.00)	34.13 (1.19)	
No	76.37 (0.02)	81.33 (0.99)	78.90 (1.00)	65.87 (1.19)	
CVD (%)					< 0.0001
Yes	16.84 (0.01)	13.82 (0.72)	15.70 (0.84)	22.73 (1.01)	
No	83.16 (0.02)	86.18 (0.72)	84.30 (0.84)	77.27 (1.01)	
Stroke (%)					0.002
Yes	5.26 (0.00)	4.17 (0.43)	5.38 (0.49)	6.68 (0.59)	
No	94.74 (0.03)	95.83 (0.43)	94.62 (0.49)	93.32 (0.59)	
Anti-hypertensive drug (%)					< 0.0001
Yes	56.81 (0.02)	47.68 (1.34)	55.75 (1.33)	71.48 (1.21)	
No	43.19 (0.01)	52.32 (1.34)	44.25 (1.33)	28.52 (1.21)	
Anti-hyperlipidemic drug (%)					< 0.0001
Yes	32.34 (0.01)	23.47 (1.11)	30.55 (1.05)	47.54 (1.25)	
No	67.66 (0.02)	76.53 (1.11)	69.45 (1.05)	52.46 (1.25)	
Anti-diabetic drug (%)					< 0.0001
Yes	19.91 (0.01)	9.05 (0.71)	11.44 (0.82)	46.66 (1.38)	
No	80.09 (0.02)	90.95 (0.71)	88.56 (0.82)	53.34 (1.38)	
All-cause mortality (%)					< 0.0001
Yes	16.80 (0.01)	15.14 (0.80)	15.17 (0.81)	21.32 (1.01)	
No	83.20 (0.02)	84.86 (0.80)	84.83 (0.81)	78.68 (1.01)	
cardio-cerebrovascular mortality (%)					< 0.0001
Yes	5.45 (0.00)	4.48 (0.40)	4.93 (0.46)	7.53 (0.61)	
No	94.55 (0.03)	95.52 (0.40)	95.07 (0.46)	92.47 (0.61)	
BMI (kg/m2)	32.98 (0.13)	32.33 (0.16)	33.04 (0.19)	33.86 (0.20)	< 0.0001
Waist circumference (cm)	110.46 (0.27)	109.19 (0.36)	109.97 (0.38)	112.92 (0.48)	< 0.0001
Income-to-poverty ratio	2.86 (0.04)	2.98 (0.05)	2.89 (0.05)	2.66 (0.04)	< 0.0001
FBG (mg/dL)	120.90 (0.60)	117.01 (0.85)	111.62 (0.59)	138.53 (1.40)	< 0.0001
FTG (mg/dL)	193.70 (2.75)	200.63 (4.19)	189.19 (3.43)	189.44 (4.87)	0.05
HbA1c	6.01 (0.02)	5.43 (0.02)	5.81 (0.01)	7.12 (0.03)	< 0.0001
Uric acid (mg/dL)	6.01 (0.02)	6.07 (0.04)	5.99 (0.03)	5.93 (0.04)	0.01
FTC (mg/dL)	198.81 (0.79)	198.49 (1.15)	203.37 (1.06)	193.39 (1.40)	< 0.0001
CRP (mg/dl)	2.12 (0.08)	1.71 (0.09)	2.07 (0.11)	2.78 (0.18)	< 0.0001

CVD, cardiovascular disease; CKD, chronic kidney disease; BMI, body mass index; FBG, fasting blood glucose; FTG, fasting triglyceride, HbA1c, Hemoglobin A1c; FTC, fasting total cholesterol; CRP, c-reactive protein.

### 3.2 Association between hemoglobin glycation index and mortality in metabolic syndrome participants

**Table 2** presents the dose-response association between HGI and mortality in people with metabolic syndrome. After all covariates being adjusted, we found no significant association between HGI and both all-cause mortality and cardio-cerebrovascular mortality, with HRs (95%CIs) of 0.99 (0.90, 1.09) and 11.12(0.93, 1.34), respectively. When exploring the relationship across tertiles of HGI, compared to T1, the HRs (95% CIs) for T2, and T3 were 0.86 (0.75, 0.98) and 0.94(0.81,1.10) for all-cause mortality. For cardio-cerebrovascular mortality, with T1 as the reference, the HRs (95% CIs) for T2, and T3 were 0.93(0.74, 1.17) and 1.01(0.78, 1.30). These results suggested a potential non-linear relationship between HGI and both all-cause and cardio-cerebrovascular mortality.

**Table 2:** Association between hemoglobin glycation index and mortality in metabolic syndrome participants.

	Model 1 HR (95% CI)	Model 2 HR (95% CI)	Model 3 HR (95% CI)
All-cause mortality			
HGI	1.37 (1.26,1.49)	1.20 (1.09,1.33)	0.99 (0.90,1.09)
HGI category			
T1	1.0	1.0	1.0
T2	1.07 (0.92,1.23)	0.87 (0.77,0.99)	0.86 (0.75,0.98)
T3	1.70 (1.47,1.97)	1.16 (1.01,1.33)	0.94 (0.81,1.10)
Cardio-cerebrovascular mortality			
HGI	1.57 (1.35,1.83)	1.44 (1.20,1.73)	1.12 (0.93,1.34)
HGI category			
T1	1.0	1.0	1.0
T2	1.17 (0.94,1.47)	0.95 (0.76,1.20)	0.93 (0.74,1.17)
T3	2.02 (1.56,2.63)	1.36 (1.06,1.74)	1.01 (0.78,1.30)

Model 1: no covariates were adjusted.

Model 2:.age, gender, race were adjusted.

Model 3:age, gender, race, education level, income-to-poverty ratio, marital status, smoking status, drinking status, vigorous recreational activity, BMI, waist,circumference stroke, CKD, CVD, hyperlipidemia, diabetes, hypertension, anti-hypertensive drug, anti-hyperlipidemic drug,anti-diabetic drug,FBG,FTG,HbA1c,uric acid,FTC, and CRP were adjusted.

**Figure 3:**
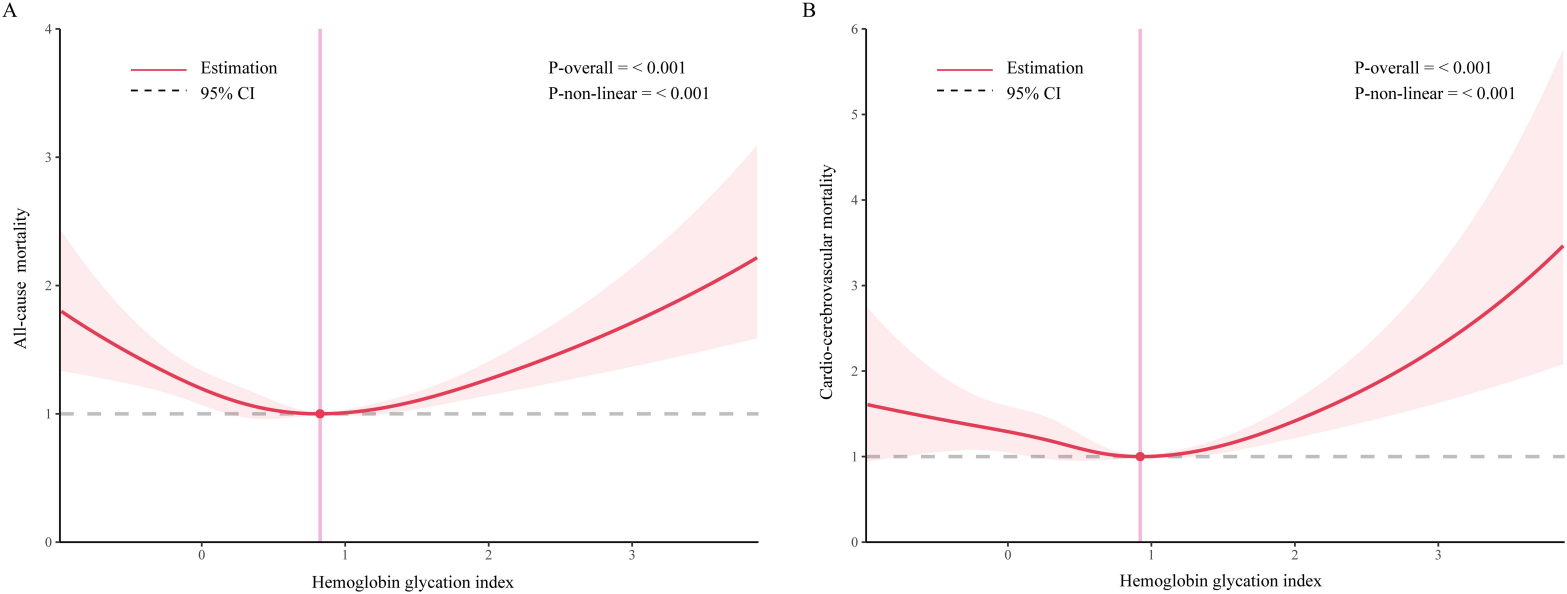
Association between hemoglobin glycation index and mortality among people with metabolic syndrome. [**(A)** all-cause mortality; **(B)** cardio-cerebrovascular mortality]. Age, gender, race, education level, income-to-poverty ratio, marital status, smoking status, drinking status, vigorous recreational activity, BMI, waist circumference, stroke, CKD, CVD, hyperlipidemia, hypertension, diabetes, anti-hypertensive drug, anti-hyperlipidemic drug, anti-diabetic drug, FBG, FTG, HbA1c, uric acid, FTC, and CRP were adjusted.

Restricted cubic splines were utilized to validate this non-linear relationship. The results indicated a U-shaped association between HGI and all-cause mortality (inflection points at 0.83) and cardio-cerebrovascular mortality (inflection points at 0.92) (**Figures 3A, B** **,** **Table 3**). Moreover, we employed segmented linear regression models to calculate the effect relationships before and after the inflection points. For all-cause mortality, the HRs (95% CIs) before and after the inflection point were 0.72 (0.63, 0.82) and 1.30 (1.17, 1.44), respectively (**Table 3**). In cardio-cerebrovascular mortality, the HRs (95% CIs) before and after the inflection point were 0.83 (0.67, 1.04) and 1.47 (1.01, 2.15), respectively (**Table 3**).

**Table 3:** Threshold effect analysis of hemoglobin glycation index on mortality in metabolic syndrome participants using a two-piecewise linear regression model.

	Adjust HR (95% CI)	P value
All-cause mortality		
Fitting by standard linear model	0.99 (0.90,1.09)	0.89
Fitting by two-piecewise linear model		
Inflection point	0.83	
< 0.83	0.72 (0.63, 0.82)	0.0023
>0.83	1.30 (1.17, 1.44)	< 0.0001
Log-likelihood ratio	<0.001	
Cardio-cerebrovascular mortality		
Fitting by standard linear model	1.12 (0.93,1.34)	0.25
Fitting by two-piecewise linear model		
Inflection point	0.92	
< 0.92	0.83 (0.67,1.04)	0.1044
>0.92	1.47 (1.01,2.15)	0.0455
Log-likelihood ratio	0.014	

Age, gender, race, education level, income-to-poverty ratio, marital status, smoking status, drinking status, vigorous recreational activity, BMI, waist circumference, stroke, CKD, CVD, hyperlipidemia, hypertension, diabetes, anti-hypertensive drug, anti-hyperlipidemic drug,anti-diabetic drug, FBG, FTG, HbA1c, uric acid, FTC, and CRP were adjusted.

### 3.3 Kaplan–Meier survival analysis

**Figure 4:**
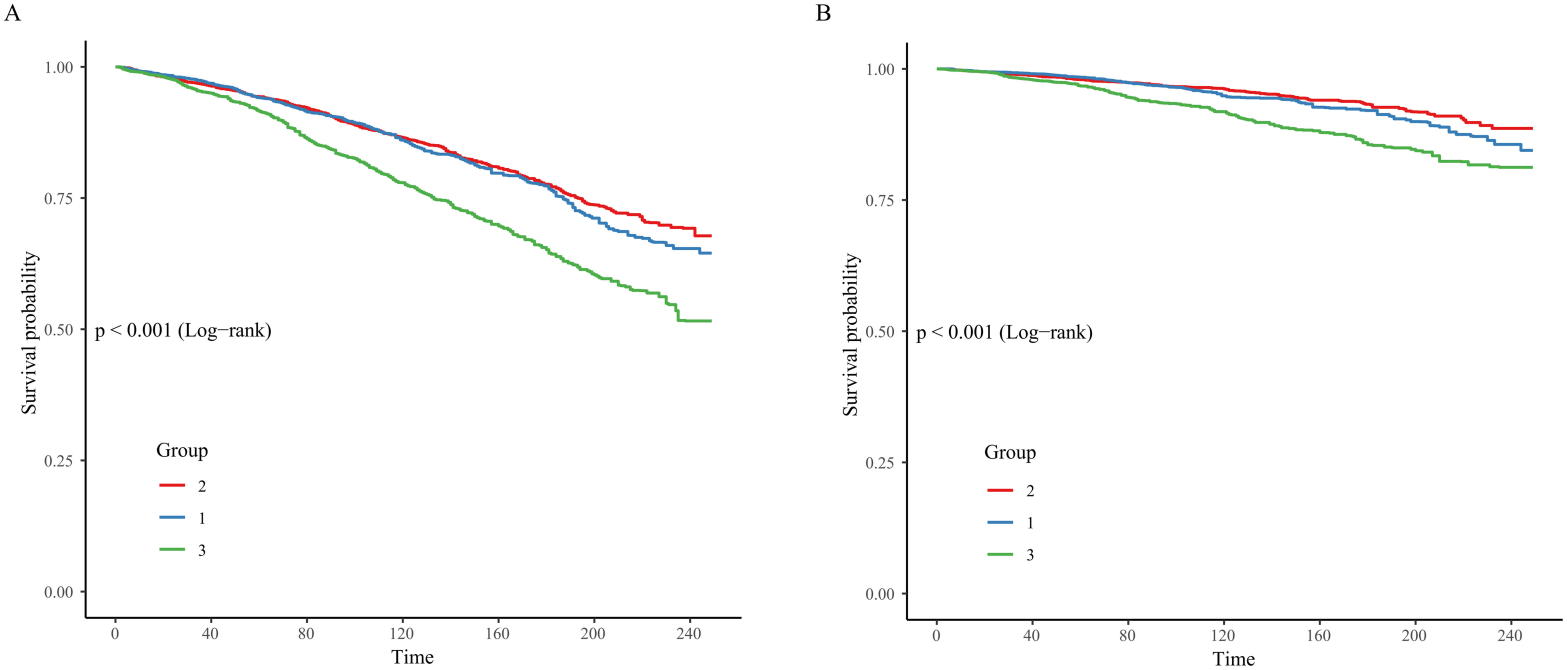
Kaplan–Meier survival analysis curves for mortality. [**(A)** all-cause mortality; **(B)** cardio-cerebrovascular mortality].

During a median follow-up period of 7.6 years (IQR, 3.9–11.6 years), 1779 deaths from all causes were recorded, with 606 attributable to cardio-cerebrovascular events. **Figure 4** illustrates the survival probability across different HGI categories. The mortality rates in these groups differed significantly among the overall population (**Figures 4A, B**). The metabolic syndrome population with HGI levels at T2 had a lower rate of all-cause mortality than the other two groups, and the same result was observed for cardio-cerebrovascular mortality.

## 4 Discussion

The association of HGI with all-cause mortality and cardio-cerebrovascular mortality in participants with metabolic syndrome was explored in this 20-year longitudinal cohort study of American adults. In the model adjusted for all covariates, no statistically significant association was found. Further analysis of linear trends suggested that the association between HGI and mortality in the metabolic syndrome population might be non-linear. Utilizing restricted cubic splines, we identified a U-shaped relationship, which might explain the lack of overall statistical significance in our findings. Compared to individuals with an HGI lower than 0.83, those with an HGI higher than 0.83 had a 58% increased risk of all-cause mortality. For cardio-cerebrovascular mortality, the risk escalated by 47% when HGI exceeded 0.92.

The KM survival analysis revealed that the population with HGI levels at T2 had the lowest risk of mortality.

In this study, both high and low HGI levels were found to be associated with an increased risk of mortality, suggesting that HGI may serve as an important clinical marker for assessing the risk of mortality in patients with metabolic syndrome. In clinical practice, this association may indicate that healthcare professionals need to pay attention to individual HGI levels when managing patients with metabolic syndrome and optimize glycemic control strategies through the development of individualized interventions, thereby effectively reducing the risk of mortality. In addition, through regular monitoring of HGI, clinicians can identify potentially high-risk metabolic syndrome patients for timely intervention to improve their long-term prognosis and provide a new basis for comprehensive treatment strategies for metabolic syndrome.

This study is the first to explore the relationship between HGI and mortality in metabolic syndrome population. Previous studies on the relationship between HGI and mortality have mostly focused on diabetes, with inconsistent results. In the AleCardio trial, elevated HGI levels correlated with heightened incidences of all-cause and cardiovascular mortality (20). Similarly, research by Sigrid et al. found that an increase in HGI was positively associated with total mortality (21). However, in the Action to Control Cardiovascular Risk in Diabetes trial, only high HGI levels were linked to all-cause mortality in diabetic adults (22). Additionally, a meta-analysis revealed a significant association between increasing HGI levels and higher rates of CVD and all-cause mortality, primarily mediated by HbA1c levels (23). Notably, a cohort study from the Netherlands in type 2 diabetic populations showed no significant relationship between HGI and total or cardiovascular mortality after adjusting for all confounding factors (24). A Chinese research demonstrated a U-shaped association between HGI and stroke, but no significant association with post-stroke mortality (25). Interestingly, in a small cohort study of individuals with coronary heart disease and diabetes, both low and high levels of HGI were found to be connected with increased mortality risk (26).

The underlying mechanisms for the U-shaped relationship between HGI and mortality risk are not fully elucidated but may involve several pathways. Low HGI might reflect a reduced rate of HbA1c, leading to prolonged hemoglobin lifespan, thereby increasing its oxidative degree and blood viscosity. This could elevate the risk of cardiovascular thrombus formation (26). On the other hand, high HGI’s mechanism in increasing all-cause and cardio-cerebrovascular mortality risk might be related to increased production of advanced glycation end-products (AGEs). AGEs, known to cause metabolic disturbances and endothelial dysfunction, have been shown to increase with higher HGI (27). Felipe et al. reported a positive correlation between HGI and AGEs in diabetic patients, possibly due to HGI enhancing the rate or process of glycation (28). Increased AGE production can lead to changes in the structure and function of vascular endothelial cells through cross-linking with extracellular proteins, resulting in reduced elasticity and increased stiffness of blood vessels. Moreover, the binding of AGEs to their receptors on endothelial cell surfaces can promote the production of pro-inflammatory factors and reactive oxygen species, inducing apoptosis of vascular smooth muscle cells (29). Additionally, AGEs contribute to the entrapment of LDL particles in the arterial walls by promoting protein cross-linking and reducing hepatic LDL clearance, thereby accelerating the development of atherosclerosis (30). Another potential mechanism involves chronic inflammation. In our study, individuals with high HGI levels often had elevated C-reactive protein levels. Previous reports have indicated a positive correlation between HGI and inflammatory markers, including neutrophils, monocytes, and C-reactive protein (31). Thus, chronic inflammation may also play a significant role in the impact of HGI on mortality. Inflammatory mediators, such as cytokines and chemokines, can alter vascular endothelial function, leading to endothelial dysfunction, reduced vasodilatory capacity, and consequently increasing the risk and poor prognosis of cardiovascular events (17, 32).

This study leverages two decades of follow-up data from NHANES to explore the relationship between HGI and mortality in individuals with metabolic syndrome, and it possesses several notable strengths. First, the comprehensiveness of the NHANES database, encompassing a diverse array of demographic characteristics such as age, gender, and race, through stringent sampling methods, enhanced the representativeness of our findings. This breadth ensured that our results more accurately reflected the overall population dynamics and trends, thereby yielding conclusions with broader applicability to the general public. Second, the large sample size intrinsic to this study served to minimize error margins, thereby bolstering the precision and statistical robustness of our findings. This aspect was crucial for the accurate analysis and interpretation of results, enhancing the scientific integrity and credibility of the study and lending greater persuasive power to its conclusions. Third, to the best of our knowledge, this research represented the longest-duration cohort study to date exploring the relationship between HGI and mortality among metabolic syndrome adults. The extended follow-up period allowed for a comprehensive assessment of the long-term impacts of specific factors on health outcomes. It provided insight into the cumulative effects of these variables over time and enriched our understanding of their prolonged influence on health. This extended observation period might offer valuable evidence for policymakers, facilitating the development of more targeted and scientifically grounded public health strategies and interventions. Fourthly, the richness of the NHANES database, with its extensive array of modifiable factors including lifestyle habits, health status, disease diagnoses, and medication usage, was a significant asset to our analysis. This comprehensive database enabled precise adjustments to be made, meticulously accounting for potential confounders. Such thorough consideration of these variables substantially enhanced the reliability and accuracy of our findings, ensuring they meet the rigorous standards expected in epidemiological research.

This study has several limitations that warrant mention. Firstly, as NHANES is an observational study, despite having long-term follow-up data, it still limited the determination of causal relationships. Secondly, the NHANES data is representative of the American population. Given the varying incidence rates of metabolic syndrome across countries, the applicability of our findings to other nations and regions remained uncertain. Lastly, while the NHANES database has comprehensively collected a variety of factors, the collection of some specific factors may not be thorough or detailed enough. This limited the consideration of these variables in the exclusion of confounding factors.

## 5 Conclusion

In this study focusing on adults with metabolic syndrome in the United States, we discovered a non-linear association of HGI with all-cause and cardio-cerebrovascular mortality. Both high and low levels of HGI were associated with an increased risk of mortality. Therefore, appropriate management of HGI levels may help in reducing the risk of death in the metabolic syndrome population. Moreover, further randomized controlled trials and cohort studies with larger samples need to be conducted to validate the stability of the findings.

## Data Availability

The datasets presented in this study can be found in online repositories. The names of the repository/repositories and accession number(s) can be found in the article/supplementary material.
